# Assessment of problem solving ability in novice programmers

**DOI:** 10.1371/journal.pone.0201919

**Published:** 2018-09-12

**Authors:** Ines Kožuh, Radovan Krajnc, Leontios J. Hadjileontiadis, Matjaž Debevc

**Affiliations:** 1 Faculty of Electrical Engineering and Computer Science, University of Maribor, Maribor, Slovenia; 2 National Education Institute Slovenia, Ljubljana, Slovenia; 3 Aristotle University of Thessaloniki, Department of Electrical and Computer Engineering, Thessaloniki, Greece; 4 Khalifa University of Science and Technology, Department of Electrical and Computer Engineering, Abu Dhabi, United Arab Emirates; Waseda University, JAPAN

## Abstract

Problem Solving (PS) skills allow students to handle problems within an educational context. PS is a core competence of Computer Science education and affects programming success. In this vein, this paper aims to investigate PS ability performance in primary school pupils of a computer course, implemented according to the Neo-Piagetian theory of cognitive development. The study included 945 Slovenian pupils, ranging from fourth to sixth grade. The effects of gender, age and consecutive years of attending the course were examined on pupils’ PS ability at the pre-operational and concrete operational stages. Pupils completed a survey questionnaire with four types of tasks (a series of statements, if-statements, loops and variables) at both stages. The analysis revealed three findings: the performance of PS ability in all tasks was, at the pre-operational stage, associated positively with performance at the concrete operational stage; there were no gender differences in PS performance at both stages, and both the grade and consecutive year of taking the computer course had an effect on PS ability performance at both stages. Those in the lowest grade and those taking the course for the first year reported lower performances than their older counterparts. These findings may help curriculum designers across the world develop efficient approaches to teaching computer courses.

## Introduction

Problem Solving (PS) has been recognised as a 21^st^ century skill due to the recent digital transformation of the economy around the globe. A common initiative of important institutions worldwide is to encourage education to focus more on the development of PS skills. The Global Agenda Council on Employment [1, 2] and the European Commission [3] thus encourage educational systems to invest more in the formation and development of PS skills in order to adapt to the current and future needs of labour markets around the world.

Practicing PS skills can be aided by learning computer programming, which is being widely promoted in primary school curricula. In 2014, European Schoolnet, a knowledge-building network of 30 Ministries of Education in Europe, revealed that 16 out of 21 European countries participating in the study had already integrated programming into their curriculum at a national, regional or local level [4]. In addition to its implementation into the primary school curriculum, it has also appeared in secondary education, where computer science courses have been adopted as a compulsory secondary school subject in many countries around the world, including in England, Australia, New Zealand, and Finland [5].

Accordingly, it is urgent for teachers to recognise the gap between the employed teaching methods and learners’ preferences and needs, and to consider how to train 21^st^ century skills [6]. In this regard, recent research has focused intensively on investigating the effects of visualised programming and teaching approaches in computer science education on students’ PS abilities and thinking [7–12] and the development of new visual environments and learning systems where students were encouraged to develop their programming abilities through PS orientation [13–15].

However, in existing research, there are a lack of studies examining the effects of the teaching of programming when following specific knowledge taxonomies, on students’ PS ability performance outcomes. This is of special importance due to the inclusion of young novice programmers into the learning process, who have only just started learning programming. In the first and second years they should gain domain knowledge and learn about the tools of programming, as well as acquiring PS strategies for simple problems. In this process, teachers have to respond to students’ reactions in the classroom efficiently and quickly. In this regard, teachers can use various well-known taxonomies for lesson planning and assessing knowledge, such as Bloom’s taxonomy [16,17], Marzano’s taxonomy [18] or SOLO Taxonomy [19]. However, teachers frequently need additional tools and methods that allow them, in an easy and structured way, to develop students’ capabilities in abstract thinking and the understanding of specific concepts. Moreover, teachers often perceive Bloom’s taxonomy to be fairly complex for monitoring the comprehension of computer science concepts among primary school students, as well as for the development of tasks and activities [20]. Despite recent advancements in programming tools and environments, the teaching of programming still remains a challenge for school teachers at various levels of education [21–24].

Accordingly, the Neo-Piagetian theory can prove invaluable in teaching programming. The reason is twofold. Firstly, Lister [25] recognised the strength of the Neo-Piagetian theory, which allows teachers to consider novice programmers’ behaviours not as a manifestation of cognitive dysfunction or mental laziness, but rather as a normal stage of cognitive development. Consequently, students’ behaviours are less exasperating for teachers and they can focus more efficiently on the teaching process. Secondly, due to its simplicity, the Neo-Piagetian theory is suitable for urgent didactic interventions when teachers, based on the recognised student’s level of abstract thinking, react with suitable questions, activities and tasks.

Even though the advantages described above prevail, the theory still has some shortcomings, such as not being directly adequate for grading [20]. Besides that, even if Gluga, Kay, Lister, and Teague [9] focused on the benefits of the Neo-Piagetian theory for classifying assessment materials, they conducted research intensively with educators. As a result, little is known about how students’ PS ability performance, which is crucial for programming, could be measured with the Neo-Piagetian theory of cognitive development. In light of this, our study attempted to investigate Slovenian primary school novice programmers’ PS ability performance according to a Neo-Piagetian approach. This may be a valuable contribution to research in the field since most of the studies regarding the global use of technology at a school level have been carried out in North America, Western Europe and Asia, while less studies have been conducted in South America, Africa, Oceania and Eastern Europe [26].

In particular, the aim of the current study is to investigate students’ PS ability performance at pre-operational and concrete operational stages, which are two of the four types of Piaget’s executive control stages. Since students were able to choose the computer course in the fourth, fifth and the sixth grades, and which could be their first, second or third time (although not third time for fourth graders), the aim was to investigate the effects of these on students’ PS ability performance at the abovementioned stages. For this purpose, the following research questions were examined:

**RQ1:** Is there a statistically significant relationship between pupils’ performance of PS ability when completing the tasks at the pre-operational stage and concrete operational stage for a series of execute commands, if-clauses, loops and variables?
**RQ2:** Is there a gender difference in performance of PS ability at the pre-operational and the concrete operational stages?
**RQ3:** What is the effect of the attending grade of the primary school on performance of PS ability at the pre-operational and the concrete operational stages?
**RQ4:** What is the effect of the consecutive year of taking the computer course on performance of PS ability at the pre-operational and the concrete operational stages?

The results of this study may be of interest to teachers and educators of programming, who create the formal teaching process and design formal instructions, accordingly. Moreover, the results may help designers of curricula for computer courses on a national scale receive feedback on the implementation of specific curricular practices, which may be shared between various educational systems.

## Background

### Problem solving skills and computer science education

PS skills refer to the capability of tackling issues and problems in different domains, such as personal, social and work. PS skills, along with critical thinking and collaborative learning skills, are viewed as a necessary part of problem-based learning [27]. When students learn through the experience of solving problems, they can learn both content and thinking strategies [28], while thinking about a complex problem or task fosters even deeper learning [29].

PS can apply to different fields, such as Psychology, Economy, Statistics, etc., and is also a core competence in computer science education [13,30–34]. Such widespread importance of PS has recently been recognised by several decisive international institutions, which emphasise investing in PS skills. For instance, the Global Agenda Council on Employment [1 p. 7, 2] reports that “policy-makers and social partners across the world have become increasingly concerned with the match between their workforces’ skills and their labour market’s needs.” Among these employees’ skills are both PS and ICT skills. Moreover, in 2016, the European Commission released A New Skills Agenda for Europe [3], where a significant lack of digital skills in Europe was recognised at all levels due to the rapid digital transformation of the economy. Almost half of the EU population lacks basic digital skills and the number of unfilled vacancies for ICT professionals is expected to almost double by 2020. Thus, European Union Member States have been called on “to invest more in digital skills`formation (including Programming/Computer Science)” [3 p. 7].

Thus, teaching programming has been incorporated into the primary school curriculum as a part of computer science education in several European countries [4]. For instance, in Slovenia, as a European country, programming has been incorporated into the national curriculum in primary schools as an optional subject and PS has been defined as one of the five main curriculum pillars.

In computer science education, various aspects of PS have been examined. On the one hand, some authors [32,35–37] write about students’ PS performance, which consists of programmers’ PS strategies and program development skills. On the other hand, Gluga et al. [9] argue that not all students are capable of a certain performance of PS, as it depends largely on their general abstract reasoning skills. Accordingly, PS ability and students’ performance measurement need to be discussed. For instance, when students’ PS ability is at the highest possible level, they solve problems in writing programs as a five-step process [9 p. 23]: “(1) Abstract the problem from its description, (2) Generate subproblems, (3) Transform subproblems into sub-solutions, (4) Recompose, and (5) Evaluate and iterate.”

### The Neo-Piagetian theory of cognitive development in computer science education

For the abovementioned reasons, in computer science education teachers have frequently a need for differentiating students according to their PS ability performance. One possible way is the Neo-Piagetian theory. It is based on the classical Piagetian theory, where a child who shows a certain level of abstract reasoning on a given problem is considered to exhibit the same level of abstract reasoning on many other problems. While the classical Piagetian theory argues that intellectual development of the individual depends on biological maturity, according to the Neo-Piagetian theory, people are thought to progress in abstract thinking regardless of their age, but rather as they gain expertise in a specific problem domain [25]. According to Piaget, students can be classified into four different groups, depending on their level of abstract thinking. They can either be at the sensorimotor, pre-operational, concrete operational or formal operational stage.

Firstly, at the sensorimotor stage, students can trace and understand the code with up to 50% accuracy. The latter means that students can understand how the program works, usually based on the teacher’s intervention questions. When tracing code, students have to put in a certain degree of effort. Instead of reading the code, they instead insert random data and try to find an answer. Secondly, at the pre-operational stage, students can trace code and conclude how the code works without any problems. However, they “tend not to abstract from the code to see a meaningful computation performed by that code” [9 p. 32]. For students, the lines in a piece of code are only weakly related. Inductive reasoning is used to derive a function of a piece of code by examining the input/output causal relationships. Thirdly, at the concrete operational stage, students are capable of routine reasoning about programming abstractions, which is limited to already known real situations and less on hypothetical situations. Moreover, students can write small programs with well-defined specifications, while they may have difficulties when writing large programs. They are also capable of deductive reasoning, which is used to derive a function just by reading the code behaviour [9]. Finally, at the formal operational stage, students can reason logically, consistently and systematically about hypothetical situations.

The abovementioned principles of the Neo-Piagetian theory can be helpful in a teaching process on a large scale. The use of the theory can be argued with constructivist approaches to programming learning where learning is seen as an active knowledge construction process. Particularly, gaining knowledge involves both learning facts and finding connections between already known and new pieces of information [15,38–41]. However, teachers are not supposed to teach knowledge, but rather to take on the role of trainers encouraging students to acquire knowledge by themselves, e.g. through problem-based learning [15].

### Effects on problem solving ability performance in computer science education

Students’ PS ability performance can be affected by gender, age and programming experience. The findings of existing research revealed that the effects of gender and age vary across levels of education. Among primary school students, Kalelioğlu [8], found a slight increment in the female students’ reflective thinking skills towards PS versus the males’ skills when learning programming, by using the code.org site. In contrast, Bruckman, Jensen, and DeBonte [42] conducted a study with 475 children and found no gender differences in programming performance when using a computer-supported collaborative environment. In higher education, male students develop programming skills easier than female students [43], while also fewer and fewer female students are attracted to computer science-related activities [44].

However, the variation in students’ ages in one class may constitute a problem for computer science teachers. For instance, according to the national curriculum for Slovenia, the computer science course is an optional subject and can be selected by pupils between the third and the sixth primary school grades (the pupils’ age is between 8 and 11 years). Since pupils attend computer courses optionally, they can attend the computer course for the first, second or third year. Consequently, teachers face the problem of heterogeneous groups of students, who could not be differentiated based on age, meaning that the year of attending the course is not a sufficient criterion since students may develop their PS abilities differently over time.

Regarding programming experience, many visual programming environments provide child novice programmers with visual support in understanding programming concepts and building codes. For instance, the Scratch environment allows pupils to engage in drag-and-drop programming, where language blocks from the block palette are dragged and attached to other blocks [45]. The pupils then receive visual feedback showing them the execution of the scripts, so that they can understand how these scripts work [46]. The Scratch environment allows students to learn “fundamental concepts such as programming logic, variables, and control structures like branching with if-then-else, looping with while constructs, and input/output capabilities, among many other things” [10 p. 151]. In addition to Scratch, a wide range of programming tools are available on the market for novice users to learn about programs, such as Hopscotch (http://www.gethopscotch.com/), Alice (http://www.alice.org/index.php), Tynker (https://www.tynker.com/) and many others.

Feng, Gardner and Feng [10] recognise two main advantages of these block-based programming environments. First, block-based languages possess a low barrier to entry, which allows students with no prior programming experience to develop the required skills easily and to stay motivated in order to continue learning. Second, these environments are suitable for various users of different ages and at different levels of programming experience, from primary school pupils and college students to professionals. However, when pupils aged 6–11 years are taught programming, Chiprianov and Gallon [11] were concerned about the idea of first learning programming through puzzles. They suggested rather giving students time to explore before teachers explain to them the visible components and functions. What is more, spatial relations can also be taught through robots.

## Related works

Previous studies have addressed PS performance, ability and related skills from various perspectives and can be classified into two groups. The first group of studies [13–15] sought to develop new visual environments and learning systems where students were encouraged to develop their programming abilities through PS orientation. The second group of studies [7–12] examined the effects of visualised programming and teaching approaches in computer science education on students’ PS abilities and thinking. In what follows, both groups of studies are discussed in detail.

### Problem solving as a core of visual programming environments and learning systems

Chao [13] developed a visual PS environment to teach programming and examined students’ patterns of computational practices (Sequence, Selection, Simple iteration, Nested iteration, and Testing), design strategies (Problem decomposition, Abutment composition, and Nesting composition) and computational performance (Goal attainment and Program size) in solving computational problems. 158 college students were included in the study and the results revealed that the students employing a more selective approach to computational practice tended to use the more advanced nesting composition, which may result in “goal attainment” computational performance.

Moreover, Chung et al. [14] proposed in their study an adaptive PS oriented system for learning programming, which can diagnose students’ learning difficulties according to the specific learning portfolios of each individual in the system. As the system classifies the learning problems, the system encourages students to understand PS correctly and, consequently, helps students develop professional programming abilities. In particular, when students solve a problem by programming code, the system diagnoses the correctness of conceptual knowledge and displays the result on the screen in real-time. Afterwards, the correctness is diagnosed by applying strategic knowledge.

Similarly, Motschnig-Pitrik, and Holzinger [15] examined similarities between student-centered teaching and new media. They defined new media as an adaptable tool that could improve the quality and effectiveness of learning and teaching. Authors saw potential in its combination with a student-centered approach, which has been shown to improve academic results and the personal values of students. The Internet can be used as a resource for acquiring knowledge and to support communication between students and the facilitator. This was named Student-Centered eLearning (SCeL). As the main characteristics of SCeL, they noted the requirement for communicative and social skills. Other requirements were: electronically available materials and the use of a computer as a resource. Student-centered teaching requires three basic conditions: the realness of the facilitator towards students, respects towards students and empathic understanding or active listening by students. In comparison to other didactic methods, student-centered teaching has the most similarities with constructivism, which considers learning to be a knowledge construction process that is built on existing knowledge, meaning that the learning is individual.

### Effects on problem solving abilities and thinking skills in computer science education

In existing research, an emphasis has been placed on investigating the effects of visualised programming and teaching approaches in computer science education on students’ PS abilities and thinking. Firstly, Lai and Yang [7] investigated the effects of visualised programming on students’ PS abilities and logical reasoning skills. One hundred and thirty pupils attending a computer course in the 6th grade of a primary school were included in the study. They were divided into two groups—the first group used Scratch and the second group used Adobe Flash to learn programming, which was also used as a tool for creating interactive content in various domains of education, such as civil engineering and medicine [47]. Before and after learning programming, students conducted a logic reasoning test. The findings revealed no statistically significant effect for Scratch programming learning on pupils’ logical reasoning. However, a significant and positive effect of Scratch programming learning on pupils’ PS ability was found. Additionally, pupils mostly showed positive attitudes towards Scratch learning, except those who demonstrated lower achievements in programming [7].

Moreover, Kalelioğlu [8] examined in his study the effects of the teaching site www.code.org on reflective thinking skills on PS. Thirty-two primary school pupils were included in the study and the data was obtained with a quasi-experiment, which was comprised of a five-week experimental process and focus groups interviews, while a reflection paper from the IT teacher was analysed as well. The findings revealed that teaching programming to primary school students in the code.org site did not cause any differences in reflective thinking skills towards PS. Female participants showed slightly higher mean scores in reflective thinking skills towards PS than male participants. Pupils were found to develop a positive attitude towards programming, and female pupils were revealed to be as successful as their male counterparts.

Secondly, the effects of various teaching approaches in computer science education on students’ PS abilities and thinking were examined. For instance, Gluga, Kay, Lister, and Teague [9] proposed the Neo-Piagetian theory for classifying materials used in learning and assessment in computer science education. An interactive web-based tutorial for Neo-Piagetian categorization of assessment tasks was described and the results were reported in an evaluation of the tutorial’s effectiveness. Twenty computer science educators participated, and the findings revealed that they classified assessment tasks with 85% accuracy in the pre-operational stage, 71% in the concrete operational stage, and 78% accuracy in the formal operational stage. After using the tutorial, self-confidence in applying Neo-Piagetian theory to classifying programming questions was higher compared to before use.

Moreover, Lye and Koh [12] reviewed 27 intervention studies to find out how computational thinking can be introduced in K-12 contexts. The authors proposed that more studies, which are focused on computational practices and computational perspectives, could be conducted in a regular classroom. When programming, the “think aloud” protocol is proposed, so that students could verbalise their thought processes, while also proposing a programming activity to be captured and analysed. In order to foster both computational practices and computational perspectives, “a constructionism-based problem-solving learning environment with information processing, scaffolding and reflection activities” is proposed [12 p. 51].

Further on, Chiprianov and Gallon [11] reported preliminary results on the implementation of the computer science course emphasising computational thinking in the mandatory national curriculum in France. Computational thinking was introduced to K-5 pupils who are between 6 and 11 years old. While children are traditionally organised into groups of about 20–30 children per class, in the computer science course it was proposed that children be organised into groups of about 10–15. The findings revealed more interaction when learning in groups of 4–5 children, than when compared to learning in pairs. Also, pupils demonstrated a need for time to explore before visible components and functions were introduced to them, while space location was initially taught through robots and, later, through puzzles.

### A critical outlook

In the above-mentioned studies, emphasis was placed on new visual environments and learning systems to support students’ PS abilities. Lai and Yang [7] advantageously found significant positive effects for learning programming in Scratch on students’ PS ability, even though Kalelioğlu [8] found no significant effect of teaching programming through the www.code.org site on reflective thinking skills towards PS. Some authors [13,14] even proposed newly developed visual programming environments, which allow students/pupils to interact constructively with visually rich elements which support the practicing of PS skills.

Moreover, research on teaching approaches towards programming advantageously revealed the positive effects of using the Neo-Piagetian theory for preparing learning materials and assessment tasks during the teaching of programming [9]. Computer science teachers selected appropriate tasks for each executive stage of students’ cognitive development with relatively high accuracy. However, overall performance of the computer course may be better when students learn in groups of 4–5 children than in pairs [11], and, with respect to gender differences, female students performed better with regard to reflective thinking skills towards PS than male students [8].

However, existing studies did not consider the Neo-Piagetian theory of cognitive development, where the authors would include students in the study and concurrently observe the effects of gender and age on students’ PS ability performance through solving particular tasks on various executive control stages. Previous studies have also not thoroughly examined why students chose the course. Also neglected were the tendencies in the years that followed the implementation of the Neo-Piagetian approach in the computer course. These issues are examined and addressed in the proposed work as described in the subsequent sections.

## Methodology

### Participants

The participants in the proposed study were 1,028 pupils from the fourth, the fifth and the sixth years attending a computer course at all public primary schools in Slovenia where this course was implemented. The Institutional Review Board (IRB) of the Faculty of Arts at the University of Maribor, Slovenia, reviewed and approved the study. An online survey questionnaire for obtaining the pupils’ response (data) was developed. After data screening, 83 units were removed from the initial dataset due to incompleteness in filling out the questionnaire, hence, questionnaire data from 945 primary school students (651 (68.9%) boys and 294 (31.1%) girls, all aged between 9 and 11 years old), were included in the analysis. Table 1 shows the pupils’ distribution across years and experience, i.e. course attendance.

**Table 1:** Distribution of pupils in the fourth, the fifth and the sixth grades in relation to attending the computer course for the first, second or third time.

Grade/year	First year	Second year	Third year	Total
**Fourth grade**	322	13	0	335
**Fifth grade**	150	208	7	365
**Sixth grade**	66	117	62	245
**Total**	538	338	69	945

### Study design and procedure

#### The computer course and the Neo-Piagetian theory of cognitive development

According to the national syllabus [48], in Slovenia, taking a computer course is not required in primary schools, but it is available to students in fourth, fifth and sixth grades who want to learn computer coding and PS thinking voluntarily. For pupils who started attending the computer course at the beginning of the school year 2016/2017, the computer course was taught for altogether 35 school hours per class in the fourth, fifth and sixth grades.

Within the above context, the implementation of the computer course adopted here was in line with the national syllabus, which consists of five main chapters [48]: Algorithms, software data, PS, as well as communication and services. The overall aims of the computer course were to:

- Learn basic computational concepts,
- Develop algorithmic/computational thinking,
- Develop PS skills,
- Realise abstraction and develop modelling skill, and
- Develop creativity, logical thinking and accuracy.

Prior to the implementation of the computer course, the teachers attended several seminars and courses about new programming tools, as well as didactic and PS strategies. Also, the principles of the Neo-Piagetian theory were introduced along with materials and tasks for pupils. At the seminars, teachers were advised to teach basic programming concepts (series of statements, if-statements, loops and variables), as well as to implement diverse activities, i.e. developing presentations, games and animations, and PS, in order to develop and upgrade pupils’ comprehension. Due to the non-homogeneity of the groups of pupils in schools, teachers had a certain degree of freedom in terms of stating individual goals for particular groups of pupils. While they were obliged to remain within the framework of the syllabus, they were able to consider the goals based on the number of pupils in the class and prior experience with pupils’ capabilities. For instance, teachers could also use different software for visual programming tools, like TouchDevelop, AppInventor or Scratch [45].

The National Education Institute Slovenia proposed the allocation of school hours and the specific guidelines for the content of problems which were to be considered at the computer course. Regarding time allocation on an annual basis, four school hours were proposed for the introduction to PS and the software Scratch. Twenty school hours were allocated to algorithms and programming where pupils were taught programming concepts. A further eight school hours were allocated for the development of the project, where pupils set their own problem, which was to be solved by using programming concepts. With regard to the specific guidelines for the content of problems, teachers were provided with access to proposed teaching and learning materials on a web platform. Among other things, it allowed teachers to find and overview tasks for each programming concept.

To sum up, in the proposed computer course, the pupils developed algorithms for simple problems, they connected multiple algorithms in the operating unit, developed programs on the algorithm basis, learned about loops, conditions and variables, identified and corrected errors in the program, learned strategies for PS, as well as appreciated the unsuccessful attempt as part of the solution, and developed perseverance. Since the students used Web applications, they also learned about safety and personal data protection.

#### Design of the evaluation session, validity and reliability

The tasks for the current study were designed according to the Neo-Piagetian theory of cognitive development that is based on Piaget’s premise that children construct knowledge when interacting with the environment. In the assimilation process, children incorporate materials from the environment into their way of thinking, while during the accommodation process their perception is changed by outer stimuli [41]. The comprehension of the previously mentioned four programming concepts and capabilities of their use was examined with respect to different levels of the Neo-Piagetian theory.

Four tasks were designed at both the pre-operational and concrete operational stage by the team of experts at the National Education Institute Slovenia. The experts have both pedagogical competencies gained during a decade of teaching experience as well as formal education in computer science. Tasks were prepared based on the findings of Gluga, Kay, Lister, and Teague [9], where the Neo-Piagetian approach was also used. Core tasks remained the same, yet the difficulty of tasks was only adjusted to the two Piagetian stages and the programming language to Scratch. For each stage, not more than one task was defined in order to ensure an enjoyable and not overly time-demanding and cognitively demanding data collection process, which is advised when children or young people are study participants [49].

A pre-test with computer science teachers was conducted to eliminate ambiguous tasks and ensure concise, valid and reliable tasks. The pre-test showed that figures should be removed from tasks, otherwise students may understand instructions differently; hence, the figures corresponding to each task were removed. Apart from that, a pilot study was constructed by administering the questionnaire to two classes, so as to ensure a flawless understanding of instructions, tasks and other relevant questions.

The tasks at the pre-operational stage were designed in a way that a pupil would be able to solve them correctly in case (s)he understood the particular programming concept (series of execute commands, if-clauses, loops and variables) and would be able to trace the code and understand its functioning.

The tasks at the concrete operational tasks were designed in a way that a pupil would be supposed to solve them in case (s)he was capable of logical operations of conservation, reversibility or transitive inference [50]. Reversible logic operations suppose that a pupil can change the program in a way that (s)he performs the same output in a different way. Since the participants in this study were primary school students who have just started with programming, the tasks were designed in a way that pupils first had to find out what the program does and then choose from a list the program that provides the same output. Most of the tasks at the concrete operational stage are based on the logical operation of conservation, while one task is based on the logical operation of reversibility.

#### Compliance with ethical standards and data collection

In the planning, execution, and reporting of the research presented in this paper, all related ethical issues were addressed. In particular, all procedures performed in this study were in accordance with the ethical standards of The Institutional Review Board (IRB) of the Faculty of Arts at the University of Maribor, Slovenia. Likewise, an informed consent form was obtained from all individual participants included in the study: parents, teachers and representatives of primary schools. Moreover, the ethical standards of the 1964 Helsinki declaration and its later amendments [51] were respected, since the study did not constitute a medical examination and presented no risk to its subjects. The research study was planned carefully, so that any potential psychological or physical harm for participants would be avoided. Furthermore, the psychological risks were reduced by not requesting participants to answer questions where the PS of particular tasks were required. Accordingly, participants were always able to answer such questions by stating that they do not know the answer or could skip tasks. Likewise, physical risks were reduced by allowing participants to stop filling out the questionnaire at any time and leave the online survey without completing the questionnaire [20]. In line with the above, the ethical standards in research released by the Society for Research in Child Development [52] and British Guidelines for research with children and young people [49] were also respected.

After attending the computer course, the pupils were given an online survey questionnaire in order to obtain data. They filled out the questionnaire in the class in the presence of their computer science teacher and were not allowed to access the online questionnaire outside of school. The access to the survey was exclusively provided to computer science teachers in all classes in 239 primary schools in Slovenia which carried out the computer course. Pupils had one academic hour (45 minutes) at their disposal to fill out the questionnaire.

### Instrument

The online survey questionnaire comprised four main sections: (1) Demographic information, (2) Tasks at the pre-operational stage, (3) Tasks at the concrete operational stage, and (4) Questions related to the computer course. In total, 13 questions were used for all the sections briefly described below.

Section 1: Demographic information included questions about gender, the grade of the primary school and the number of years taking the computer course. Students could attend either the fourth, fifth or the sixth grades of primary school and could take the computer course either for the first, second or for the third time, independent of the grade they were attending. The only impossible combination was to be in the fourth grade and attend the computer course for the third time. The aim of identifying the pupils’ history of taking the computer course was to identify pupils’ prior program experience [53].

Sections 2 and 3: Programming performance was measured by providing tasks at the pre-operational and the concrete operational stages. At both stages, the tasks covered four main elements: A series of execute commands, if-clauses, loops and variables. For each of these elements two tasks were provided: one at the pre-operational and one at the concrete operational stage.

Altogether there were eight tasks and their order was randomised in order to reduce the learning effect. Similarly, participants did not know at which of the abovementioned stages they were. Participants were asked to respond to each close-ended question/task by choosing one of the five provided answer options, while only one answer was correct.

Section 4: Computer course-related questions were presented to the pupils regarding the reason for taking the computer course. Students were provided with one semi-closed question, so that they were able to select the reason from a list or write down their own reason. Moreover, pupils were asked about their aspirations of taking the computer course in the following year. It was measured with one semi-closed question, so that pupils were allowed to select any of the listed answer options or were invited to list their own thoughts.

A detailed description of the questionnaire is included in S1 Appendix, while particular tasks can be seen in S1–S11 Figs. At the end of the questionnaire there is a note explaining how questions with tasks are connected to the Neo-Piagetian stages.

### Data analysis

Descriptive statistics were used to describe pupils’ performance of PS ability at the pre-operational and concrete operational stages. In order to compare PS ability performance in various tasks and at both stages, a Chi-Square test for associations was conducted. To further reveal whether there was a difference between boys and girls, the Mann-Whitney U Test was used, since the assumptions for the use of this test were met, since there was a non-normal distribution of data; the dependent variable was measured at the continuous level, the independent variable consisted of two categorical, independent groups, and the assumption of independence of observations was met. Further on, when inspecting whether pupils’ PS ability performance at the pre-operational and the concrete operational stages was affected either by the pupils’ grade or the number of years taking the computer course, the Kruskal-Wallis H test was used. Before statistical analyses, the normality of data was tested using the Kolmogorov-Smirnov test for the RQ2-RQ4, where testing the normality was one of the assumptions for the particular statistical test to be used. The analyses were performed with the IBM SPSS Statistics 21.0 (https://www.ibm.com/analytics/us/en/technology/spss/). Data is available in S1 Dataset.

## Results

### RQ1: Pupils’ PS performance across stages

To answer RQ1, the descriptive statistics, when summing up correct and incorrect answers of each task per stage, resulted in pupils’ performance of PS with a median value of 2.00 (SD = 1.30, min = 0, max = 4) for the pre-operational stage and a median value of 1.00 (SD = 1.24, min = 0, max = 4) for the concrete operational stage.

Table 2 shows the results from the Chi-square test regarding the existence or not of statistically significant differences in the PS performance of a single type of tasks between the pre-operational and concrete operational levels. When completing the task of series of execute commands, a statistically significant association between pupils’ PS ability performance at the pre-operational and concrete operational stages exists, i.e., *x*^2^ (1,945) = 19.09, *p* < .001.

**Table 2:** Chi-square comparison table of pairs of students for series of execute commands, if-clauses, loops and variables.

Tasks	Pre-operational stage	Concrete operational stage				*x*^*2*^ *(1)*	*p*
		Incorrect		Correct			
		Count	%	Count	%		
**Series of execute commands**	**Incorrect**	309	53.74	145	39.19	19.09	0.00*
	**Correct**	266	46.26	225	60.81		
**If-clauses**	**Incorrect**	390	63.83	103	30.84	94.20	0.00*
	**Correct**	221	36.17	231	69.16		
**Loops**	**Incorrect**	414	67.43	147	44.41	47.23	0.00*
	**Correct**	200	32.57	184	55.59		
**Variables**	**Incorrect**	364	66.42	109	27.46	139.83	0.00*
	**Correct**	184	33.58	288	72.54		

* p < 0.01

Table 2 further shows that there was a statistically significant association between PS ability performance at the pre-operational and concrete operational stages when completing tasks of if-clauses, *x*^2^ (1,945) = 94.20, *p* < .001. Table 2 also shows a statistically significant association between performance at the pre-operational and concrete operational stages when completing tasks of loops, *x*^2^ (1,945) = 47.23, *p* < .001. Likewise, the same association was found when completing tasks for variables, *x*^2^ (1,945) = 139.83, *p* < .001.

### RQ2: Gender differences on PS performance at each stage

Before performing a gender analysis, the Kolmogorov-Smirnov (with Lilliefors correction) test was applied to the data to test for normality. From the latter, it was found that the data were not distributed normally (*p* < .001). The results of the gender analysis revealed no statistically significant effect of gender on performance of PS at the pre-operational stage (*p* > .05) or the concrete operational stage (*p* > .05).

Moreover, an analysis was performed as to whether gender differences were evident in scores for each task at the pre-operational and concrete-operational stages. The results revealed statistically significant differences between boys and girls only in scores for the task of loops at the pre-operational stage (*U* = 85.55, *z* = −3.07, *p* < .01). Boys had an average rank of 488.58, while girls had an average rank of 438.50.

### RQ3: Effects of pupils’ grade on PS performance at each stage

Regarding the RQ3, the adopted Kruskal-Wallis H test showed that there was a statistically significant difference in PS ability performance at the pre-operational stage, *x*^2^ (2) = 17.78, *p* < .001, with a mean rank performance of 435.29, 469.62, and 529.59 for the fourth to the sixth grades, respectively. Pairwise comparisons using the Kruskal-Wallis test (*p* = .05) revealed that pupils in the fourth grade demonstrated lower PS ability performance at the pre-operational stage than pupils in the sixth grade. Similarly, pupils in the fifth grade demonstrated lower scores than those in the sixth grade. However, there was no difference in PS ability performance at the pre-operational stage between fourth graders and those in the fifth grade.

Likewise, the Kruskal-Wallis H test showed a statistically significant difference in PS ability performance at the concrete operational stage, *x*^2^ (2) = 31.54, *p* < .001, with a mean rank performance of 417.87, 476.70, and 542.87 for the fourth to sixth grades, respectively. Pairwise comparisons using the Kruskal-Wallis test (*p* = .05) revealed statistically significant differences between all groups of pupils. Accordingly, sixth graders demonstrated the highest mean ranks in PS ability performance at the concrete operational stage, followed by those in the fifth and fourth grade.

### RQ4: Effects of pupils’ consecutive computer course attendance on PS performance at each stage

Kruskal-Wallis H test results revealed statistically significant differences in PS ability performance at the pre-operational stage, *x*^2^ (2) = 19.40, *p* < .001, with a mean rank performance of 444.99, 495.85, and 579.45 for the first to third consecutive years of computer course attendance, respectively. Pairwise comparisons with the Kruskal-Wallis test (*p* = .05) further showed that students who attended the computer course for the first time were worse in their PS ability performance at the pre-operational stage compared to those who attended the course for the second or third year. However, there was no difference in PS ability performance at the pre-operational stage between pupils who attended the course for the second and third years.

The analysis was repeated for the concrete operational stage. The Kruskal-Wallis H test showed statistically significant differences in PS ability performance at the concrete operational stage, *x*^2^ (2) = 26.38, *p* < .001. The resulted mean rank performance was 435.44, 515.82, and 556.15 for those who attended the course for the first to third consecutive years of computer course attendance, respectively. Moreover, pairwise comparisons with the Kruskal-Wallis test (*p* = .05) revealed that first-year pupils demonstrated the lowest PS ability performance at the concrete operational stage compared to the second- and third-year pupils. However, no difference was found in PS ability performance at the concrete operational stage between the second- and third-year pupils.

### Pupils’ thoughts on the computer course

Finally, pupils’ reasons for taking the computer course were explored. The majority (72.8%) took the course due to their own interest in programming, 6.5% decided to attend the course due to their parents’ advice, 4.1% due to their fellow pupils, and the rest of the pupils attended the course due to teachers’ advice, because they like the teacher, or for some other reason. No statistically significant results were found when analysing whether taking the computer course due to students’ interest or any other reason had an influence on students’ PS ability performance at both stages. However, a Chi-Square test revealed a statistically significant association between boys and girls who decided to attend the computer course due to their own interest in the computer course or not, *x*^2^ (1,945) = 12.12, *p* < .001, shown in Table 3. As one can see, more girls and boys decided for the course due to their own interest than for any other reason. Further on, more boys than girls counted in both types of reasons, also due to the demographic structure of the sample.

**Table 3:** Chi-square comparison table of pairs of pupils according to gender and reason for taking the computer course.

		Reason for taking the computer course				*x*^*2*^ *(1)*	*p*
		My own interest		Other reason			
		Count	%	Count	%		
**Gender**	**Male**	496	72.09	155	60.31	12.12	0.00*
	**Female**	192	27.91	102	39.69		
**Total**		688	100	257	100		

* p < 0.001

Moreover, pupils’ tendency to take the computer course in the forthcoming year was examined as well. The majority (48.1%), reported that they would take the computer course once again, 35.3% would not take it and the rest of the answers were undefined. The relationship was also examined between the tendency for taking the computer course or not in the forthcoming year and the reason for taking it for the current year. Altogether, 789 students were included in this analysis, and the applied Chi-Square test revealed a statistically significant association, *x*^2^ (1,789) = 17.17, *p* < .001, as shown in Table 4. The latter shows that more of those who decided to attend the course for their own interest in the current year tended to attend the course also in the forthcoming year compared to those who took the course for some other reason.

**Table 4:** Chi-square comparison table of pairs of students according to tendency and reason for taking the computer course.

		Tendency for taking the computer course				*x*^*2*^ *(1)*	*p*
		yes		no			
		Count	%	Count	%		
**Reason for taking the computer course**	**My own interest**	362	79.56	222	66.47	17.17	0.00*
	**Other reason**	93	20.44	112	33.53		
**Total**		455	100	334	100		

* p < 0.001

## Discussion

### Delving into the findings

The aim of this study was to investigate PS ability performance in primary school pupils in Slovenia who attended a computer course where the Neo-Piagetian approach was used. PS ability performance was measured at the pre-operational and concrete operational stages, where the effects of gender, the pupils’ grade and year of taking the computer course were observed.

The analysis revealed three main findings:

1. The performance of PS in tasks or series of execute commands, if-clauses, loops and variables at the pre-operational stage was associated positively with performance at the concrete operational stage. It indicates that those who demonstrate good PS ability-performance scores at the lower executive control stage, similarly demonstrate better scores at the higher stage. Based on that, the development of pupils’ PS abilities can be understood as progressive, going through sequential executive cognitive control stages.
2. When inspecting for gender differences in PS ability performance at both the pre-operational and the concrete operational stages, no gender differences were found either at the pre-operational or concrete operational stages. These findings may show, intriguingly, that boys do not advance in cognitive thinking abilities faster than girls when problem tasks are being solved at both the executive control stages. So far, as no effect of gender at the pre-operational stage is notable, these findings are in line with the findings of previous studies where no gender differences were found in programming performance [42] or computational thinking skill levels [54]. However, the findings of the study presented in this paper revealed that boys demonstrated a higher level of PS ability performance than girls when solving the task of loops at the pre-operational stage. Accordingly, in this type of task, boys seem to overtake girls, which is in contrast to the findings of Kalelioğlu [8], where girls demonstrated higher mean scores in reflective thinking skills towards PS than boys. These discrepancies in the findings may result from various factors, such as the domain knowledge pupils acquire, cognitive abilities, effects from the social environment, and the attitudes developed towards programming. These findings can also be explained further with the situation later in life when the number of female pupils interested in the computer science activities decreases [44] and male pupils at the tertiary education level develop programming skills with more ease than female pupils [43].
3. Pupils in the sixth grade demonstrated better performance than those in the fourth and fifth grades at both stages, while no significant differences were found between pupils in the fourth and those in the fifth grades. The findings even showed the significant effect of consecutive years of taking the computer course, where the findings intriguingly showed significant differences in PS ability performance only between the first- and the second-, as well as the first- and third-year pupils, but not between the second- and third-year pupils. Accordingly, the findings of our study complement the results of Liu, Zhi, Hicks, and Barnes [53] where pupils’ PS behaviours were found to be related to their self-reported prior programming experiences. Moreover, our findings also show, intriguingly, that there was a significant effect of pupils’ age on PS ability performance. This issue can be understood with the principle of the classical Piagetian theory, which argues that students’ cognitive abilities depend on their biological maturity. However, the Neo-Piagetian theory does not consider age to be a precondition for students’ progress in abstract thinking. In our case, this phenomenon could be understood from three different aspects. The first aspect may be an insufficient number of school hours for the computer course students attended. Only 35 school hours may not be enough for there to be obvious effects of the teaching process, where the differentiation of pupils based on their stage of cognitive development occurred. The second aspect may be that, due to the newly introduced teaching approach, following the Neo-Piagetian theory, teachers may have had initial difficulties in differentiating pupils based on their stages of cognitive development, which may have led to differentiating based on pupils’ age. Since the computer course is an optional subject, computer course classes are very heterogeneous groups of students with various levels of pre-knowledge and PS abilities. The third aspect may be the possible effects of the social environment, where upbringing, family and social network may play an important role.

Accordingly, the results of the current study complement the findings of Gluga, Kay, Lister, and Teague [9] where the Neo-Piagetian approach was proposed as well. While these authors conducted a study with educators, proposed a freely available tutorial and substantiated that it can be used efficiently by educators when classifying assessment materials, in the current study this approach was used when preparing tasks for pupils to measure PS ability performance. Consequently, the findings presented in this paper enhance the findings of Gluga, Kay, Lister and Teague [9], since we have pointed out further how the learnable Neo-Piagetian approach to classifying assessment tasks could be practically used in the classroom when PS ability performance is being assessed.

Likewise, through pupils’ tendency to also select the computer course in the forthcoming year, it can be recognised that the majority of primary school pupils developed a positive attitude towards programming during the computer course, which is in line with the findings of Lai and Yang [7], where sixth-grade pupils were included in the study. As of now, pupils mostly decide for programming due to their own interest. However, those who are advised by parents or other counterparts are in the minority, which may indicate a group of pupils who are not familiar with programming to a great extent or have a lower interest in programming. In this case, the findings of Chiprianov and Gallon [11] may be relevant, where the authors suggested teaching programming through robots first and, later, through puzzles, which is the case in learning with Scratch, so that we avoid overly difficult beginnings.

### Implications and limitations

The findings of the present research may have a wide range of implications for practice. The first major practical contribution of the present research is that the findings contribute to educators of programming and indirectly to pupils as well [20]. According to the findings of the current study, where the Neo-Piagetian theory of cognitive development was used, it can help educators, when recognising that a particular group of pupils did not complete the task at the pre-operational stage correctly, to conclude that students are at a sensorimotor stage of thinking. As a result, these pupils cannot follow the code and do not understand the specific concept correctly. This may be a sign for the teacher to develop activities which would help students reduce mistakes in understanding the structures of computer science concepts. Likewise, when pupils do not complete the tasks at the concrete-operational stage, they are likely to be at the sensorimotor or pre-operational stage. Such pupils are not able to think abstractly and may refuse an explanation with flowcharts. In this case, a teacher could develop activities in which pupils could explain the purpose and get a sense of the functioning of the already developed code, whereby they could recognise its abstractness, which is a precondition for being capable of writing more complex software where abstractness is necessary. The advantage of a teaching approach with the Neo-Piagetian theory is that it does not require that the order of stages of abstract thinking be respected. As a result, students can write software before they can follow the code or even describe it. When an assessment of students is held, exams based on Neo-Piagetian theory can help teachers recognise at which stage students are, so that teachers can respond appropriately.

Secondly, the findings may help designers of the curriculum for the computer course on a national level obtain feedback on the efficiency of current pedagogical approaches, and may be of further help since, due to these findings, it will be easier to track consequences in students’ gained knowledge due to any alterations in the curriculum which could occur in the following years. For instance, if a computer course would be implemented in the third grade of primary school, it would be interesting to see whether these students achieved better coding performance in the fourth-grade when compared to their predecessors.

Thirdly, the current findings contribute to popular debates about the importance of programming and computational thinking, where students learn how to deal with large problems by breaking them down into smaller, more manageable parts. Programming is viewed as the literacy of today and a help to practice “21st century skills such as PS, team work and analytical thinking” recognised and vastly supported by the European Commission [55] as well. The reason is the current situation on the labour market, as “more than 90% of professional occupations nowadays require digital competences, including programming” [55].

The present study has three main limitations. The first limitation is that the results of the study could have been affected by pupils’ prior experiences with programming. As the computer science course is an optional subject, pupils may have had various levels of pre-knowledge of programming. Also, pupils may have had various prior experience with programming outside of school, due to their special interest in self-initiative programming learning.

The second limitation stems from the self-reporting used in the study. Since an online questionnaire, filled out in the classroom, was used, it is not known to what extent participants answered the questions honestly and to what extent the measured PS ability is accurate. Hence, we suggest that authors of future studies combine quantitative research methods with qualitative ones, so that qualitative data would elaborate and explain the findings of the collected quantitative data.

The third limitation is the high number of teachers included in teaching the computer courses in different primary schools. While there is a common syllabus in use on a national level which provides recommendations for the implementation of a course, there are still possibilities that there are differences in teaching across teachers, which may influence the results. Consequently, it is not known to what extent the teaching performance influenced students’ programming performance. The results could be different if all students were taught under the same conditions, e.g. by e-learning.

## Conclusion and future work

The main purpose of this research study was to make a scientific contribution to understanding whether there exist effects of gender, grade and consecutive year of attending the computer course on students’ PS ability performance at two Neo-Piaget’s executive control stages. The findings revealed, firstly, that students’ performance of PS ability at the pre-operational stage was statistically significantly associated with performance at the concrete operational stage when students were completing tasks of series or execute commands, if-clauses, loops and variables. If lower performance at the pre-operational stage was demonstrated, the performance was lower at the concrete operational stage as well. Secondly, no statistically significant effect of gender on PS ability performance at both stages was found, except for in the case of loops, where male students demonstrated higher scores than female students. Thirdly, a statistically significant effect was found for the pupils’ grade and the number of consecutive years taking a computer course on their PS ability performance. Particularly, pupils in the sixth grade demonstrated better performance than those in the fourth and fifth grades at both stages, while pupils also attending the computer course for the third time demonstrated higher scores compared to those attending the course for the first or the second time.

In the future, it would be intriguing if every school year the study would be repeated, so that the programming performance of pupils could be compared across all three grades and, concurrently, those who attend the computer course for the first, second and third times. As a result, increments in programming performance could be measured across years and for every class in primary schools in Slovenia. Moreover, future research would benefit from measuring the performance of PS before and after taking the computer course among primary school pupils, so that comparisons could be made and the direct effects of the implementation of the computer course could be measured.

Based on the presented findings, there is a recognised necessity for novel approaches to teaching programming where the heterogeneity of students in classrooms is considered, especially in terms of their PS abilities. The Neo-Piagetian theory allows teachers to consider students in an appropriate way, while domain knowledge and the time available for learning programming still appears to be important. Only when teachers are capable of classifying students based on their PS abilities, domain knowledge and practicing, can PS skills be sufficiently provided, which may be beneficial for society in the long term, so that the goals of 21st century education will be reached and needs of the labour market satisfied.

## Supporting information

S1 Appendix Questionnaire. (DOCX)

S1 Dataset Dataset of collected data. (SAV)

S1 Fig The task with the pencil. (TIF)

S2 Fig The figure of program A. (TIF)

S3 Fig The figure of programs B, C and D. (TIF)

S4 Fig The task with steps. (TIF)

S5 Fig The figure of program A. (TIF)

S6 Fig The figure of programs B, C and D. (TIF)

S7 Fig The task with moving the figure. (TIF)

S8 Fig The figure of program A. (TIF)

S9 Fig The figure of programs B, C and D. (TIF)

S10 Fig The task with playing “meow” sound. (TIF)

S11 Fig The task with variables “points” and “lives”. (TIF)
