# Supplementary material for: Assessment of problem solving ability in novice programmers
Source: PLoS One. 2018 Sep 12;13(9):e0201919. doi: 10.1371/journal.pone.0201919 (PMC6135368; doi:10.1371/journal.pone.0201919)
Supplement: S1 Appendix — (DOCX) [file pone.0201919.s001.docx]

S1 Appendix. Questionnaire.

1. **I am attending:**
2. The fourth grade.
3. The fifth grade.
4. The sixth grade.
5. **Gender:**
6. Boy.
7. Girl.
8. **I attend the computer course this year:**
9. For the first time.
10. For the second time.
11. For the third time.
12. **Why did you choose the computer course?**
13. My parents advised me to.
14. A teacher or other staff at my school advised me to.
15. Other classmates chose this course, too.
16. I like the teacher of the computer course at my school.
17. I am interested in computer course.
18. Other reason (please, specify): _______________
19. **Will you choose the computer course in the following school year as well?**
20. Yes.
21. No.
22. I do not know.
23. **The figure draws a line when the pencil is put down. How long a line is drawn by the figure when we click on the green flag?**

<S1 Fig about here>

**S1 Fig. The task with the pencil.**

Select the correct answer.

1. 30
2. 50
3. 20
4. 100
5. I do not know.
6. **In program A, the figure is moving and goes ‘meow’.**

< S2 Fig about here >

**S2 Fig. The figure of program A.**

< S3 Fig about here >

**S3 Fig. The figure of programs B, C and D.**

In which program (B, C or D) does the figure make the same number of steps and go ‘meow’ equally as in program A?

Select the correct answer.

1. In none of the listed programs B, C or D.
2. In the program B.
3. In the program C.
4. In the program D.
5. I do not know.
6. **How many steps make the figure when the program executes?**

< S4 Fig about here >

**S4 Fig. The task with steps.**

Select the correct answer.

1. 200 steps
2. 150 steps
3. 100 steps
4. 50 steps
5. I do not know.
6. **We made a program for a figure cat.**

< S5 Fig about here >

**S5 Fig. The figure of program A.**

< S6 Fig about here >

**S6 Fig. The figure of programs B, C and D.**

In which program (B, C or D) does the figure go ‘meow’ equally as in the program A?

Select the correct answer.

1. In none of the listed programs B, C or D.
2. In the program B.
3. In the program C.
4. In the program D.
5. I do not know.
6. **We want to move the figure for 100 steps. What value do we need to set in variable Nr. to make that happen?**

< S7 Fig about here >

**S7 Fig. The task with moving the figure.**

Select the correct answer.

1. 10
2. 40
3. 45
4. 100
5. I do not know.
6. **In which program (B, C or D) does the figure make the same number of steps as in the program A?**

< S8 Fig about here >

**S8 Fig. The figure of program A.**

< S9 Fig about here >

**S9 Fig. The figure of programs B, C and D.**

Select the correct answer.

1. In none of the listed programs B, C or D.
2. In the program B.
3. In the program C.
4. In the program D.
5. I do not know.
6. **We click on a green flag. About how many seconds after clicking does the cat go meow?**

< S10 Fig about here >

**S10 Fig. The task with playing “meow” sound.**

Select the correct answer.

1. After 5 seconds.
2. After 10 seconds.
3. After 15 seconds.
4. After 6 seconds.
5. I do not know.
6. **What are the values of the variables “points” and “lives”, when all commands are executed?**

< S11 Fig about here >

**S11 Fig. The task with variables “points” and “lives”.**

Select the correct answer.

1. Points = 0 and Lives = 10
2. Points = 1 and Lives = 8
3. Points = 2 and Lives = 5
4. Points = 3 and Lives = 5
5. I do not know.

**Note:** *Questions 6, 8, 12 and 13 represent the tasks at the pre-operational executive stage and questions 7, 9, 10 and 11 represent the tasks at the concrete operational Neo-Piaget’s executive control stage.*
