# Supplementary figures and images for: Assessment of problem solving ability in novice programmers
Source: PLoS One. 2018 Sep 12;13(9):e0201919. doi: 10.1371/journal.pone.0201919 (PMC6135368; doi:10.1371/journal.pone.0201919)

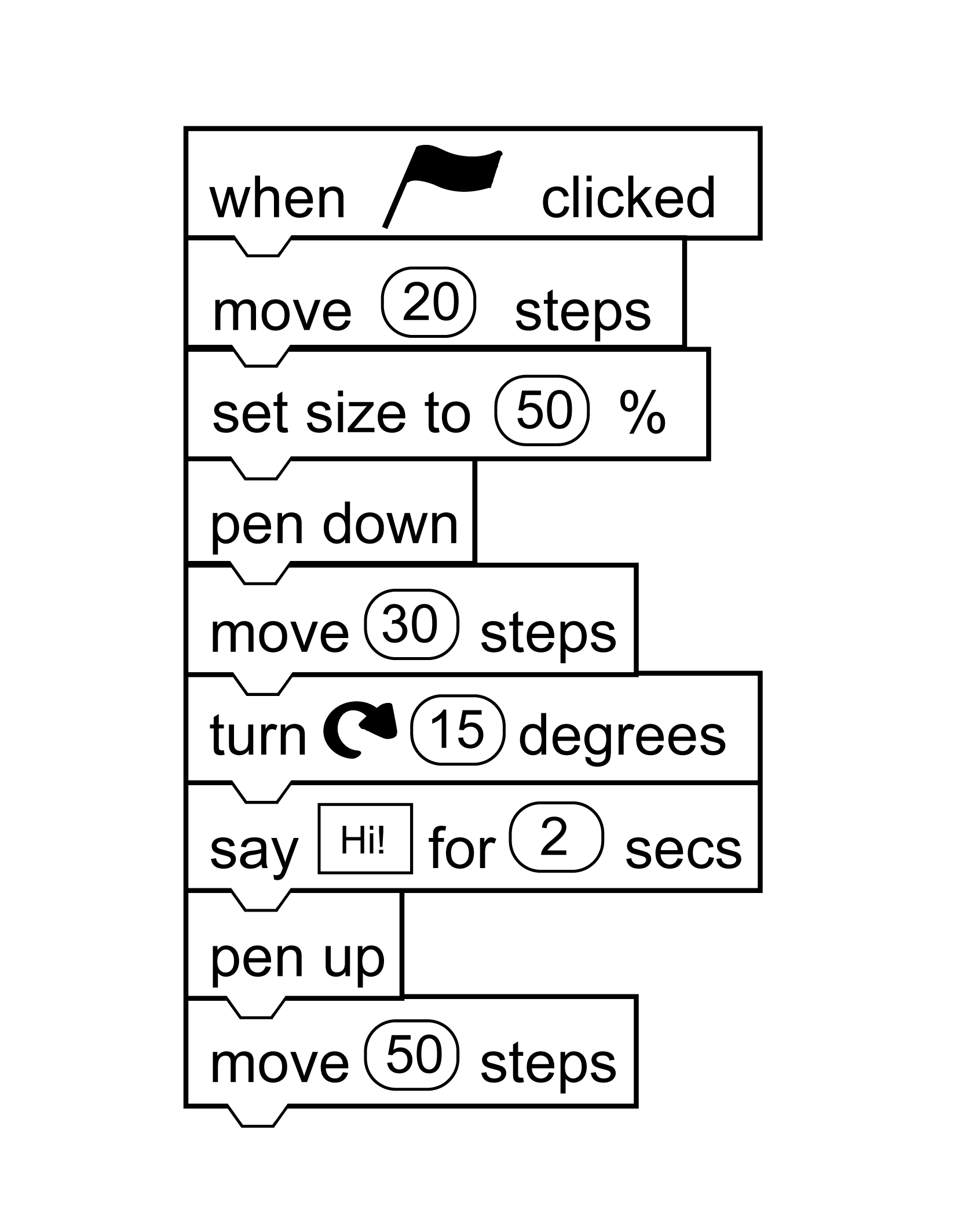

Supplement: S1 Fig — (TIF) [file pone.0201919.s003.tif]

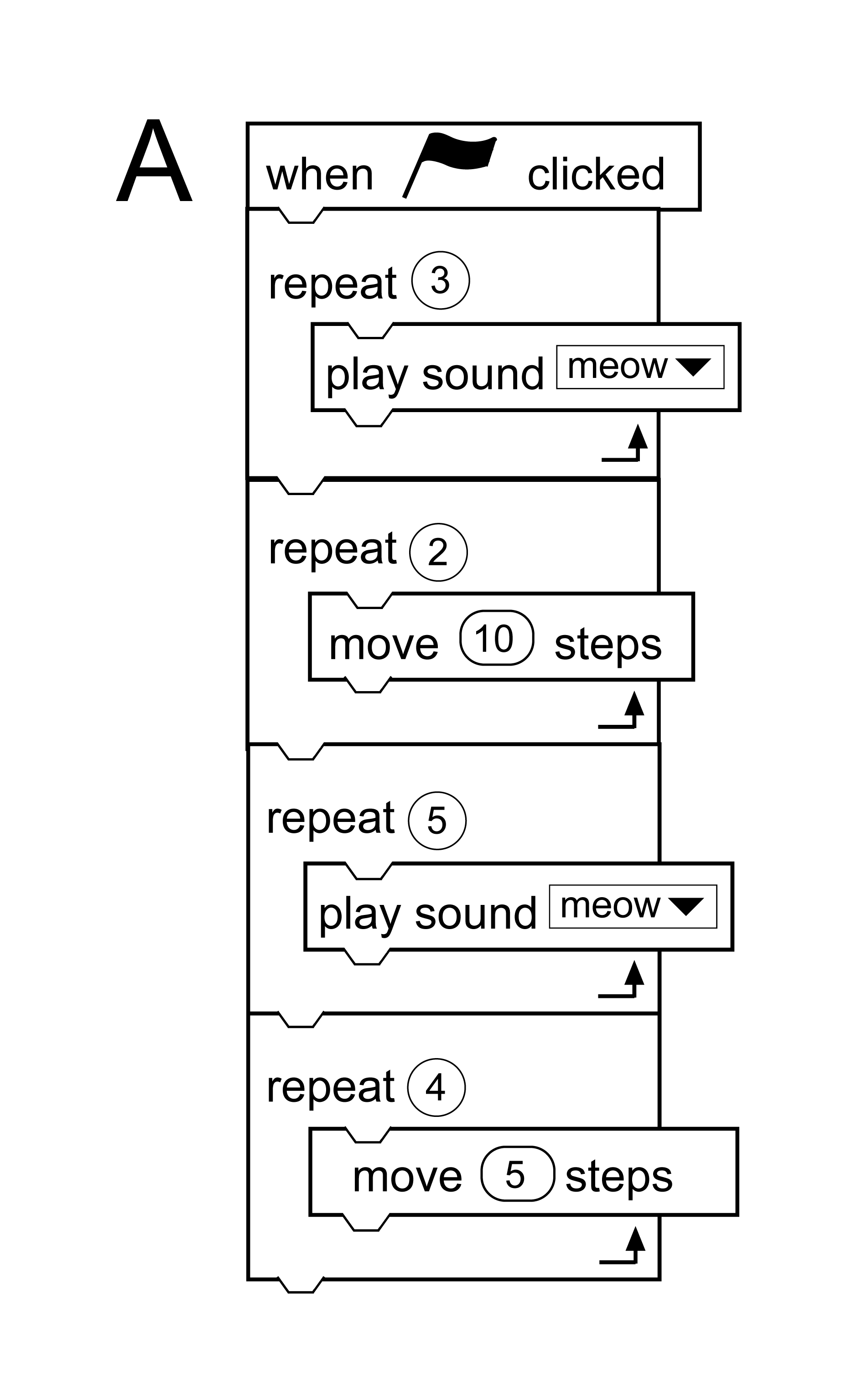

Supplement: S2 Fig — (TIF) [file pone.0201919.s004.tif]

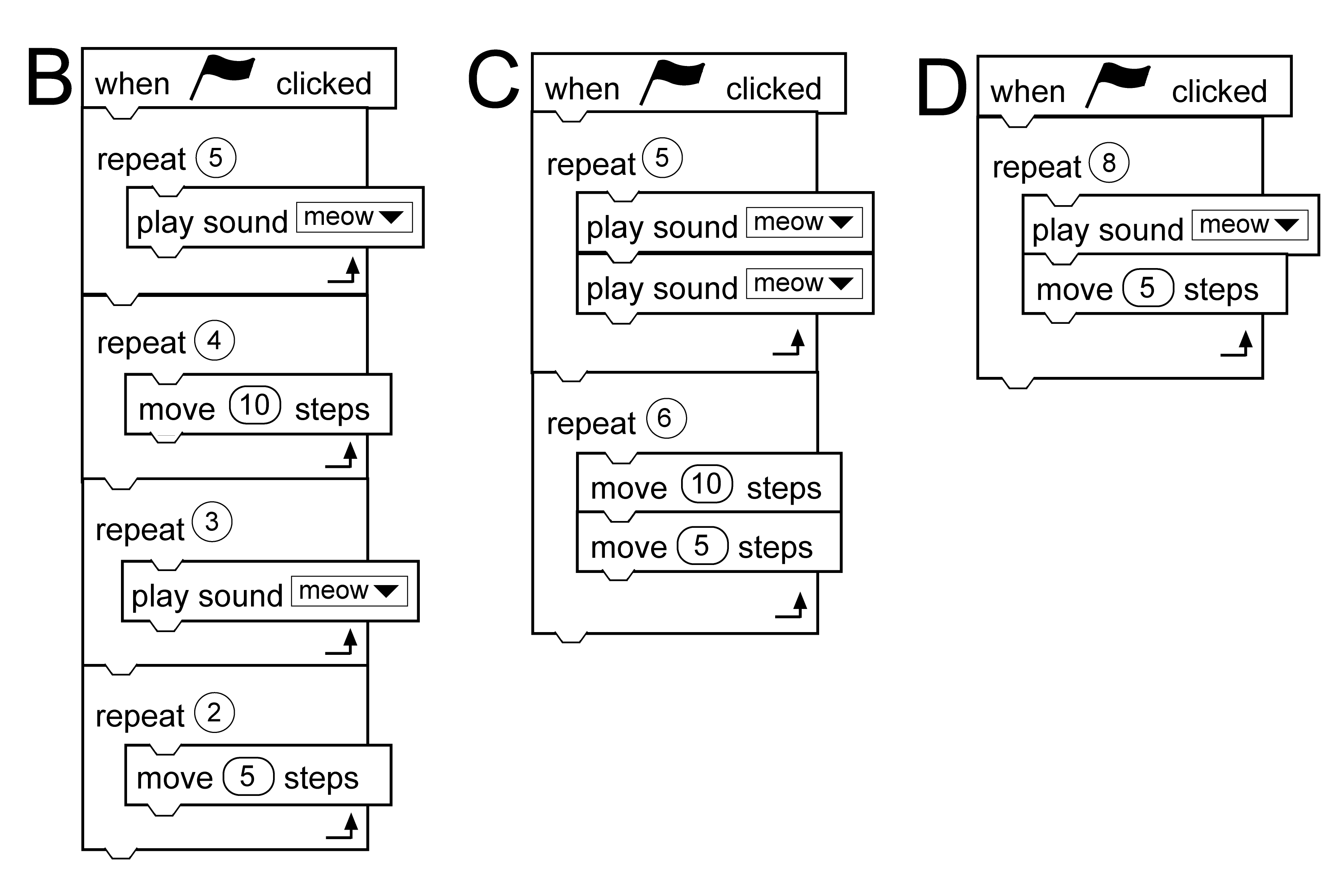

Supplement: S3 Fig — (TIF) [file pone.0201919.s005.tif]

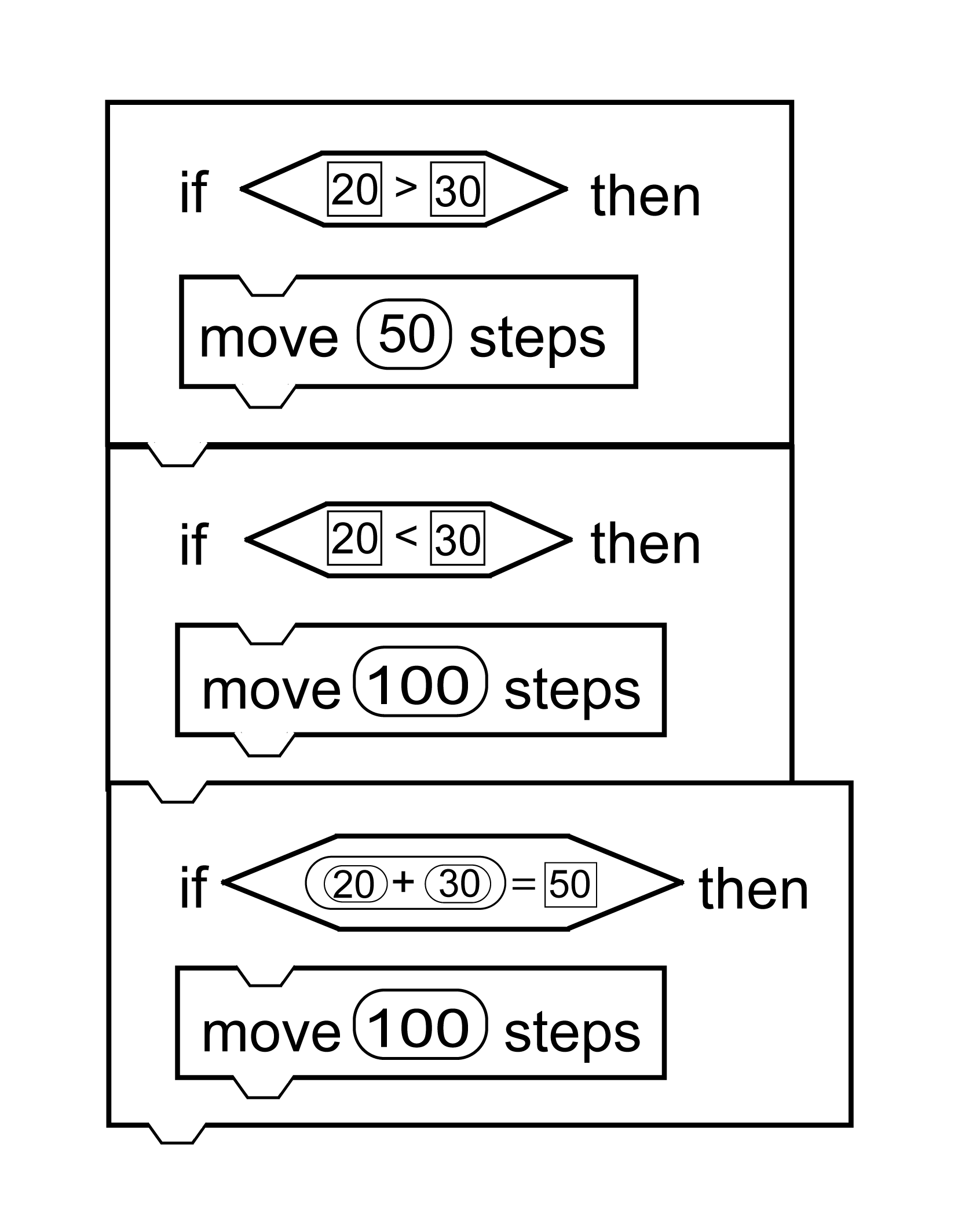

Supplement: S4 Fig — (TIF) [file pone.0201919.s006.tif]

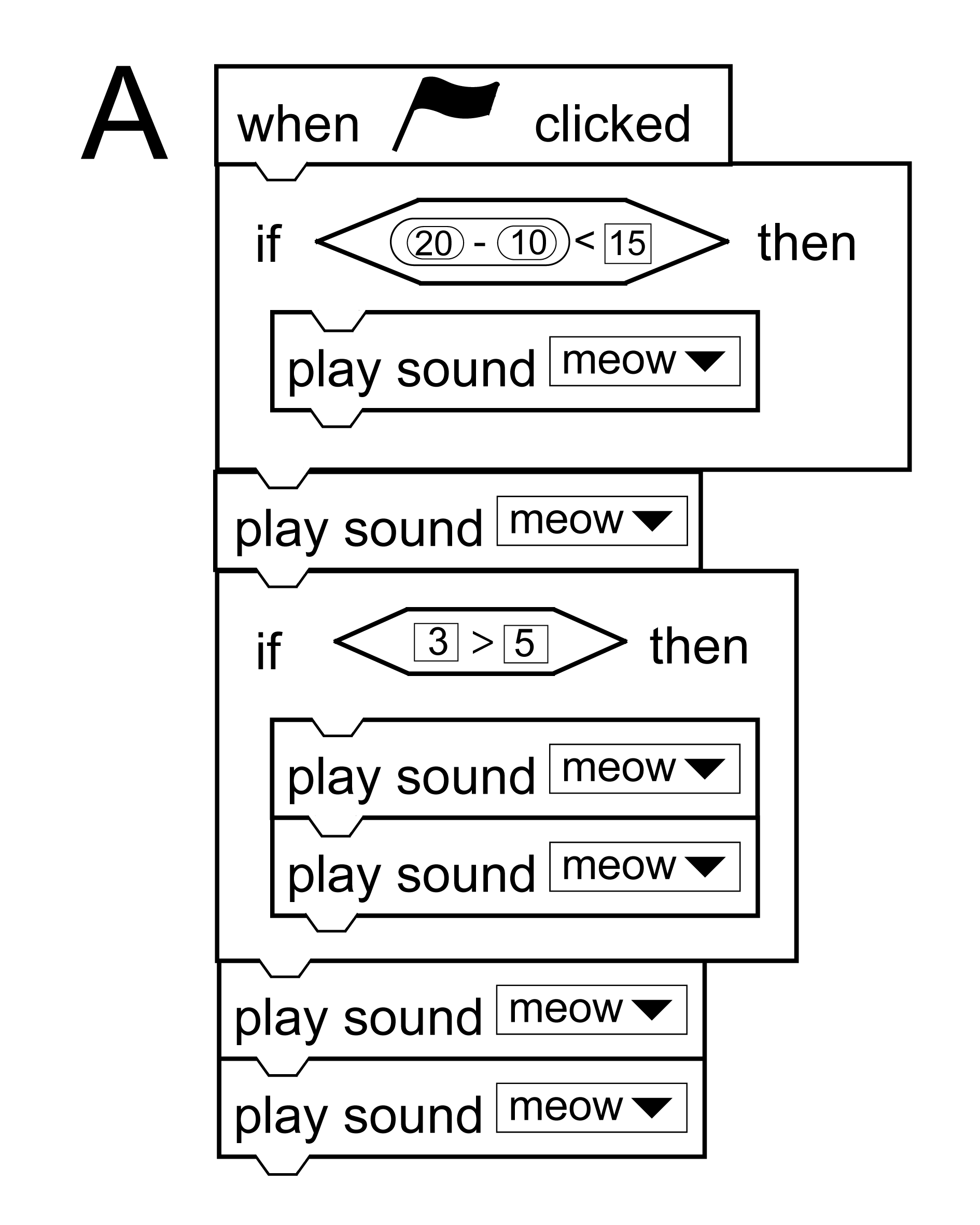

Supplement: S5 Fig — (TIF) [file pone.0201919.s007.tif]

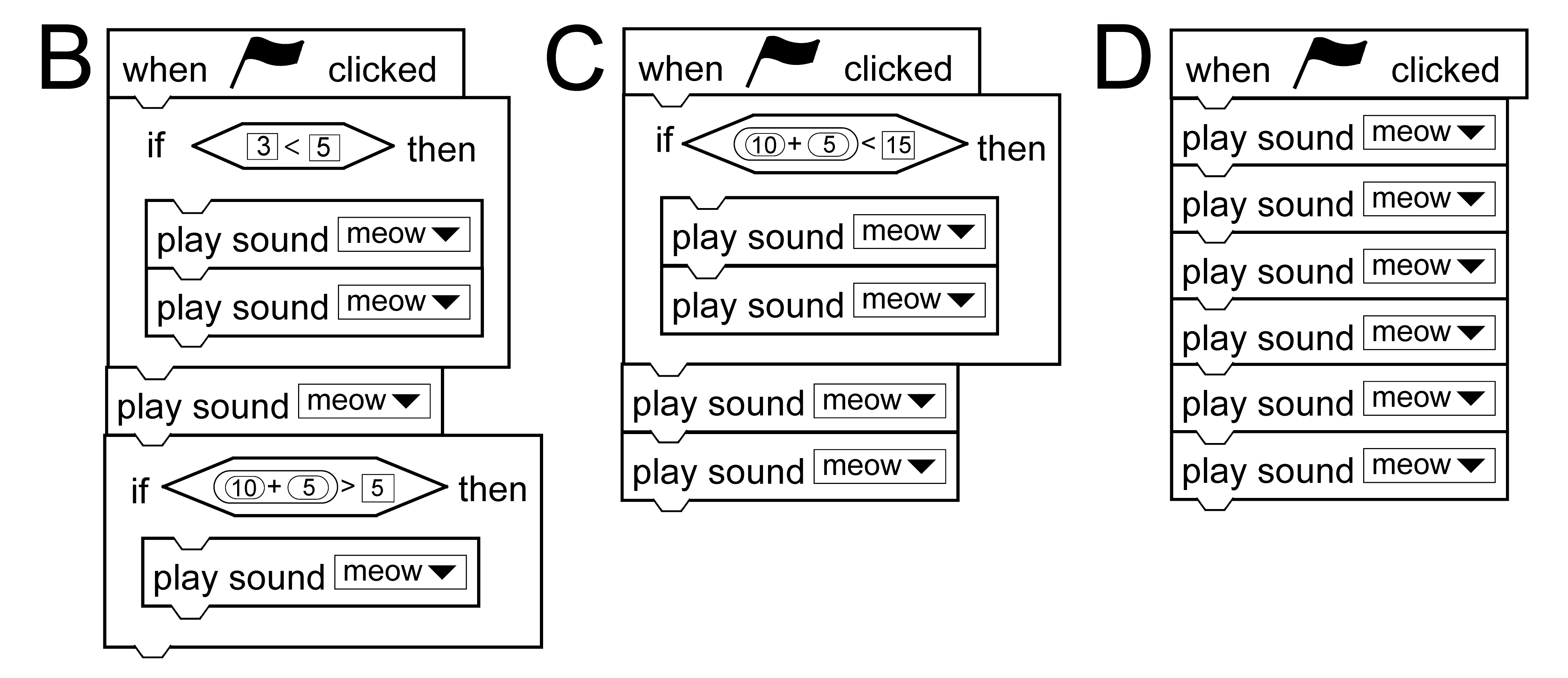

Supplement: S6 Fig — (TIF) [file pone.0201919.s008.tif]

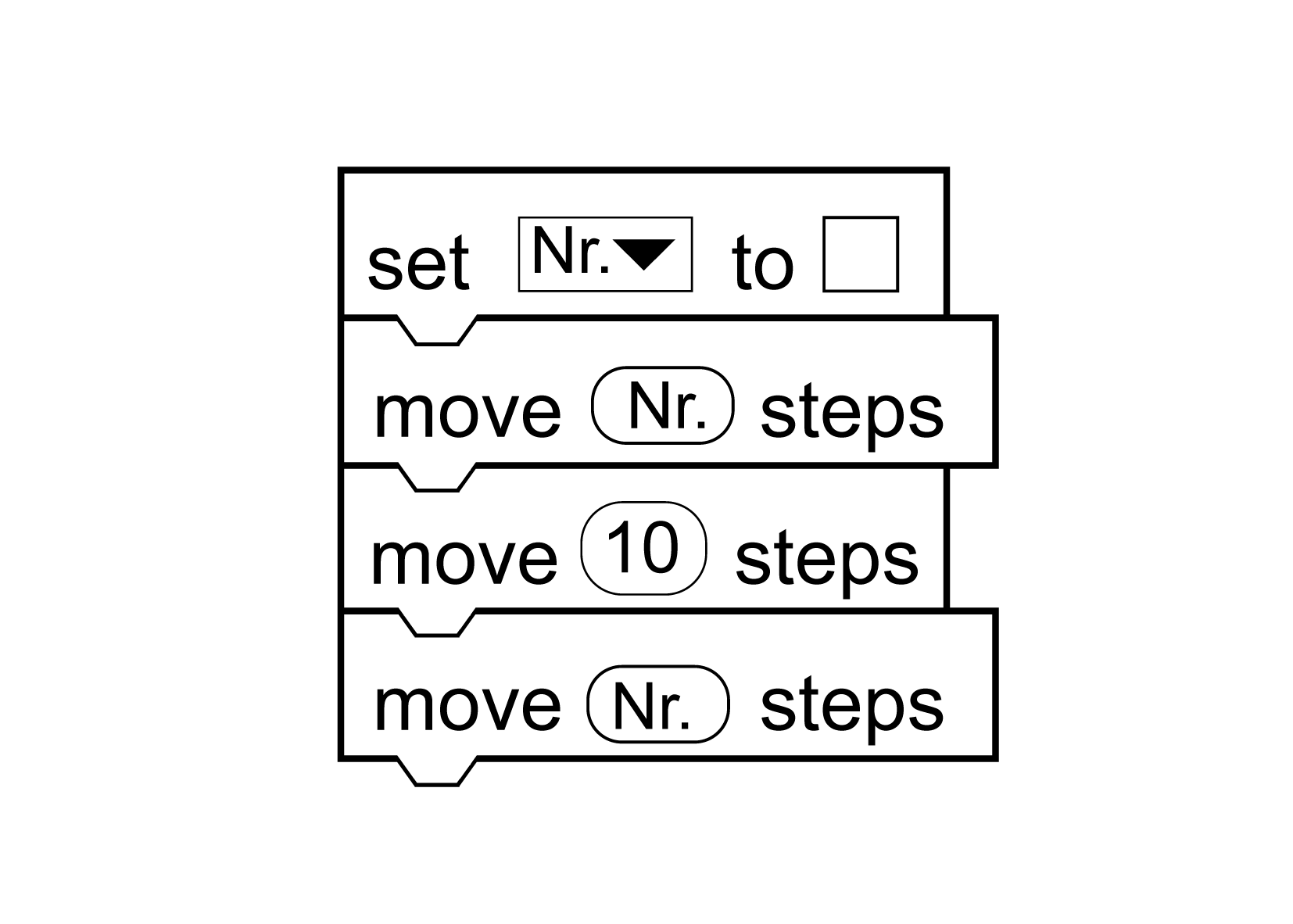

Supplement: S7 Fig — (TIF) [file pone.0201919.s009.tif]

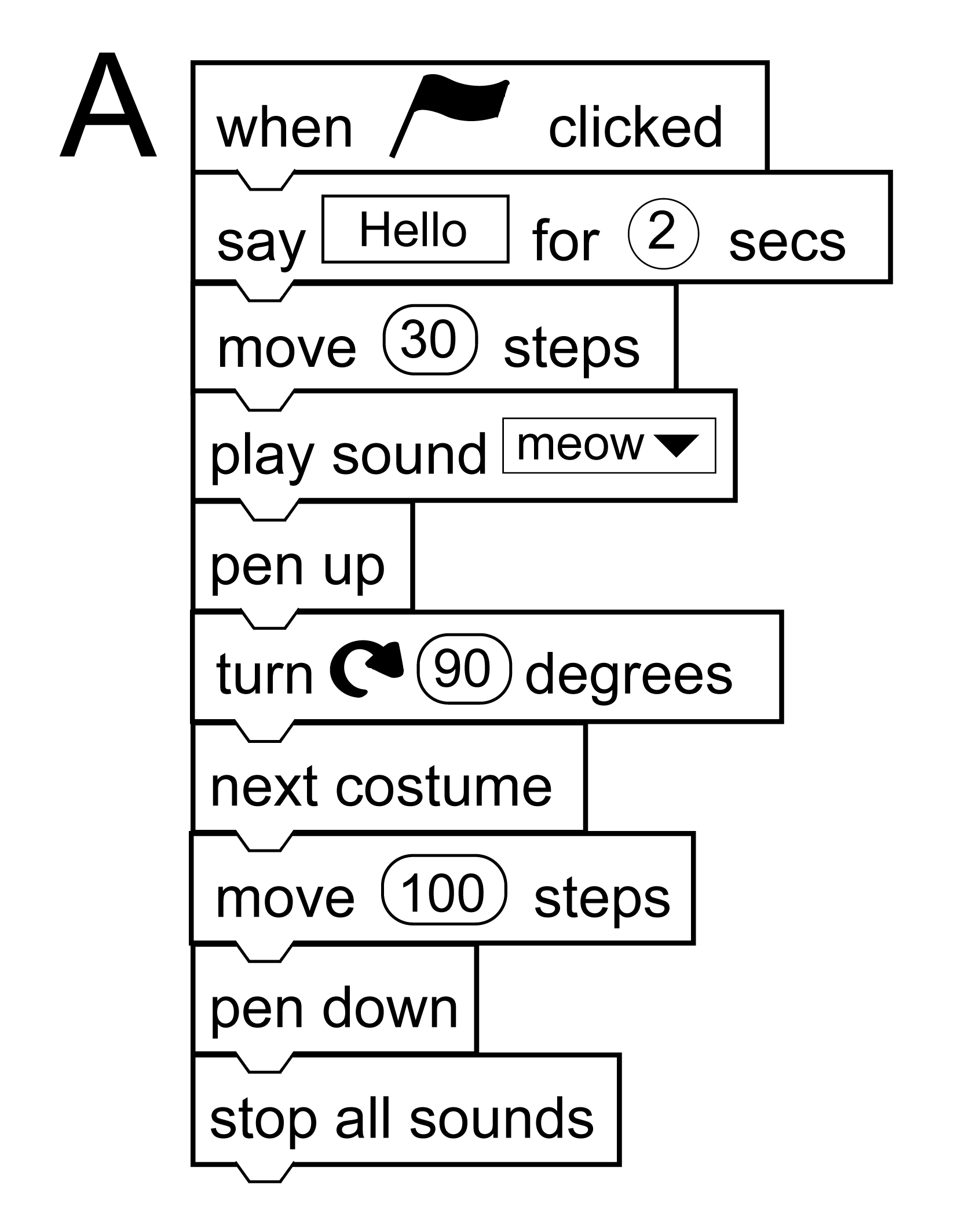

Supplement: S8 Fig — (TIF) [file pone.0201919.s010.tif]

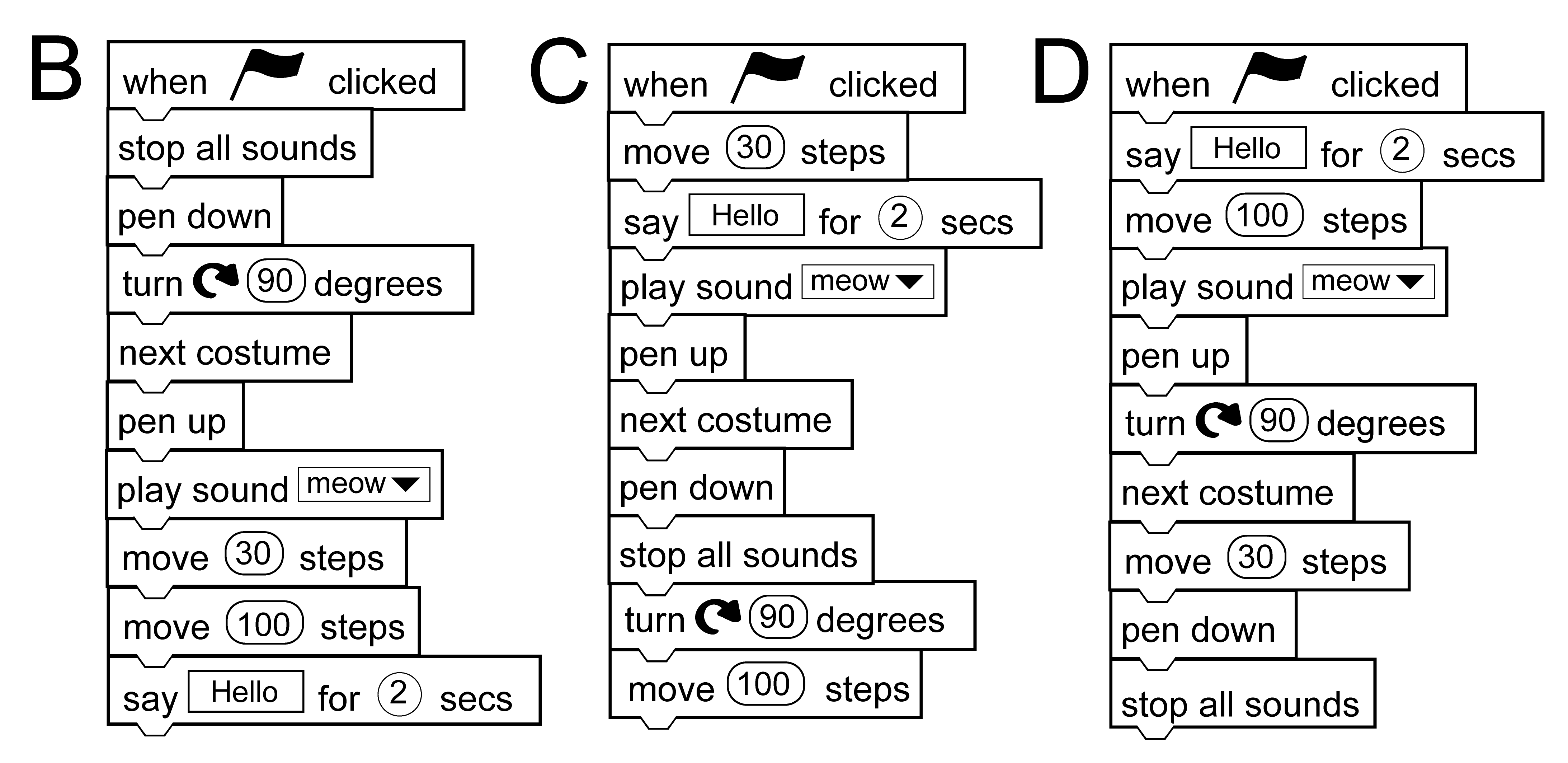

Supplement: S9 Fig — (TIF) [file pone.0201919.s011.tif]

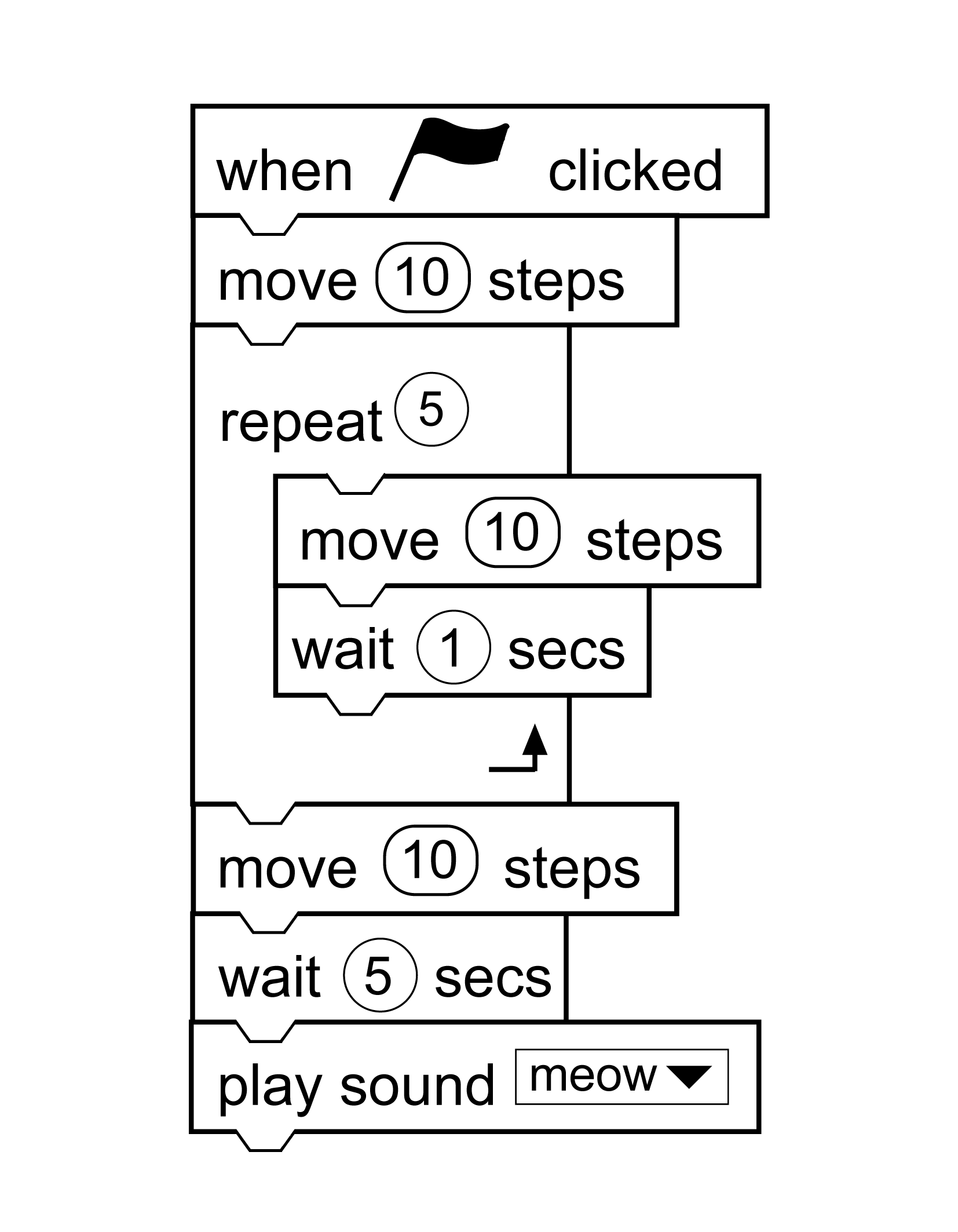

Supplement: S10 Fig — (TIF) [file pone.0201919.s012.tif]

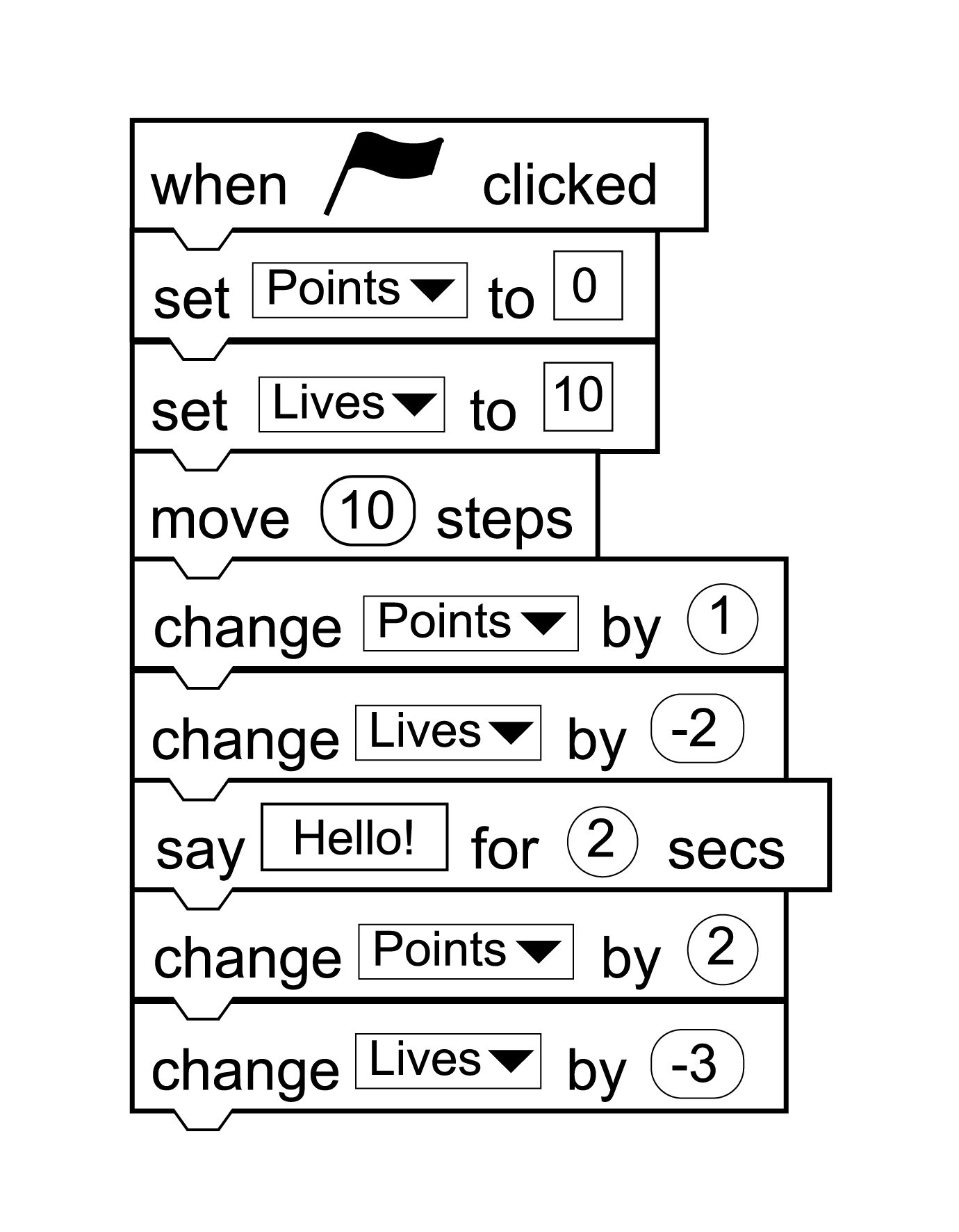

Supplement: S11 Fig — (TIF) [file pone.0201919.s013.tif]
